# Nutritional Metabolomics: Postprandial Response of Meals Relating to Vegan, Lacto-Ovo Vegetarian, and Omnivore Diets

**DOI:** 10.3390/nu10081063

**Published:** 2018-08-10

**Authors:** Millie Rådjursöga, Helen M. Lindqvist, Anders Pedersen, B. Göran Karlsson, Daniel Malmodin, Lars Ellegård, Anna Winkvist

**Affiliations:** 1 Department of Internal Medicine and Clinical Nutrition, Sahlgrenska Academy, University of Gothenburg, Box 459, 405 30 Gothenburg, Sweden; helen.lindqvist@gu.se (H.M.L.); lasse.ellegard@nutrition.gu.se (L.E.); anna.winkvist@nutrition.gu.se (A.W.); 2 Swedish NMR Centre, University of Gothenburg, Box 465, 405 30 Gothenburg, Sweden; anders.pedersen@nmr.gu.se (A.P.); goran.karlsson@nmr.gu.se (B.G.K.); daniel.malmodin@nmr.gu.se (D.M.)

**Keywords:** metabolomics, nutrition, NMR, human metabolome, postprandial, serum, vegan, vegetarian, omnivore, diets

## Abstract

Metabolomics provide an unbiased tool for exploring the modulation of the human metabolome in response to food intake. This study applied metabolomics to capture the postprandial metabolic response to breakfast meals corresponding to vegan (VE), lacto ovo-vegetarian (LOV), and omnivore (OM) diets. In a cross over design 32 healthy volunteers (16 men and 16 females) consumed breakfast meals in a randomized order during three consecutive days. Fasting and 3 h postprandial serum samples were collected and then subjected to metabolite profiling using ^1^H-nuclear magnetic resonance (NMR) spectroscopy. Changes in concentration of identified and discriminating metabolites, between fasting and postprandial state, were compared across meals. Betaine, choline, and creatine displayed higher concentration in the OM breakfast, while 3-hydroxyisobutyrate, carnitine, proline, and tyrosine showed an increase for the LOV and unidentified free fatty acids displayed a higher concentration after the VE breakfast. Using ^1^H NMR metabolomics it was possible to detect and distinguish the metabolic response of three different breakfast meals corresponding to vegan, lacto-ovo vegetarian, and omnivore diets in serum.

## 1. Introduction

Human health is related to lifestyle factors, including diet, level of physical activity, body weight management, and smoking [1]. In present times of climate change and the discussion of the environmental impact of humans, vegan and vegetarian diets have become of interest in a wider population. In this light, the effects on health of diets excluding animal products have become further important to understand. 

Dietary intake of red and processed meat has been associated with increased risk to develop type 2 diabetes, obesity, cardio vascular disease, and cancer [2,3]. However, lifestyle factors other than diet also affect health outcomes, i.e., body mass index (BMI), smoking, stress, alcohol use, level of physical activity, and mental health [1]. These characteristics may differ between individuals that are habitually consuming omnivore and vegetarian diets, respectively. They may be difficult to statistically adjust for and thus confound associations between diet and health in observational studies, and this may explain why results of the health effects of different diets are conflicting [4,5,6]. Here, diet interventions are important. 

Metabolite profiling has proven to be useful in identifying dietary perturbations from both diets and single meal challenges [7]. The use of controlled dietary trials has the promise to validate potential biomarkers that are identified in epidemiological studies, and several studies have investigated the short term metabolic response to different diets [8,9,10]. However, to our knowledge, no studies investigating the metabolic response to meals relating to omnivore or vegetarian diets in healthy individuals have been performed.

The measure of postprandial responses after a meal challenge may promote the understanding of the metabolic fate of individual foods, biological mechanisms involved, and the relationship to different diets [11,12]. In this respect, meal challenges and postprandial metabolomics measurements hold the potential to complement the understanding of the metabolic response to different foods and diets in healthy and diseased individuals [13]. In addition, diet cross-over interventions, where subjects act as their own controls, reduce variance in relation to physiological variation between individuals, lifestyle factors, and reporting bias, and they therefore provide an opportunity to evaluate the metabolic effects from the diet intervention alone.

In the present cross-over study the metabolic response of meals corresponding to vegan (VE), lacto-ovo vegetarian (LOV), and omnivore (OM) diets in healthy individuals were investigated. Our aims included the characterization of metabolic profiles from the different breakfast meals and the identification of metabolites increasing or decreasing in concentration in response to the three meals. 

## 2. Materials and Methods 

### 2.1. Ethical Approvement

The project was approved by the Regional Ethical Review Board in Gothenburg (reference number 561-12) and was registered with ClinicalTrials.gov (identifier: NCT02039596). 

### 2.2. Study Participants

Volunteers were recruited in August to October 2013 by way of advertisement at the University of Gothenburg, Sweden, and Chalmers University of Technology in Gothenburg, Sweden. Before entering the study participants gave written informed consent.

In total, 32 healthy subjects, 16 males and 16 females, were enrolled in the study (Table 1). Volunteers were assessed for suitability by screening, which included a short lifestyle questionnaire, a three-day weighed-food diary, clinical chemistry tests including blood hemoglobin levels, serum electrolytes, iron status, vitamin B12 and folate, calcium, magnesium, creatinine, liver transaminases, bilirubin and alkaline phosphatase, C-reactive protein, plasma glucose, and thyroid status. Anthropometrical measurements, such as height, weight, and body composition were also included in the screening. Body composition was measured with bioimpedance (ImpediMed Bioimp Version 5.3.1.1,Carlsbad, CA, USA). Volunteers were considered healthy if they had normal clinical chemistry, were apparently healthy and with no regular use of medications (contraceptives were permitted), and BMI >18.5 and <30 kg/m^2^. 

Exclusion criteria included: pregnancy or lactation, use of nicotine, dietary supplements, natural remedies and/or herbal tea, alcohol consumption higher than five units per week (1 unit = 12 g alcohol), allergies to food items included in the study, unwilling to consume foods included in the meals, and the practice of an extreme diet or intent to change physical activity and/or dietary habits before or during the intervention. 

### 2.3. Study Design

Study participants consumed one out of three breakfasts; vegan (VE), lacto-ovo vegetarian (LOV), and omnivore (OM) in a randomized order during three consecutive days. Breakfasts were consumed at the test kitchen of the Department of Internal Medicine and Clinical Nutrition, University of Gothenburg, Sweden, between 07:30 and 09:30 h. VE consisted of tea with oat-milk, soya based yoghurt containing blueberries, two rye bread sandwiches, one with lentil mash and green pepper topping, and one with cashew butter and banana. LOV consisted of tea with milk, fruit yoghurt (raspberry/rhubarb), two rye bread sandwiches, one with cottage cheese and apple, and the other with hard cheese and tomato. OM consisted of tea with milk, boiled egg and caviar, two rye bread sandwiches, one topped with ham and red pepper, and one with liver pâté and cucumber (Table 2). Study participants could freely choose large (750 kcal) or small (500 kcal) breakfast size; all but two females and one male chose the small breakfast. Composition of both breakfast sizes are found in supplementary material Tables S1–S3 and macro nutrients content in Table S4. Briefly, amounts of carbohydrates, fat, and proteins for the small breakfast were 74 g, 17 g, and 19 g, respectively, for VE, and 60 g, 22 g, and 24 g for LOV and 60 g, 26 g, and 26 g for OM. In addition, the fiber content of the VE, LOV, and OM breakfasts were 9.9 g, 6.9 g, and 5.8 g, respectively. 

To help stabilize the background metabolic profiles, a standardized evening meal was provided to be consumed between 18:00–20:00 h on the evening before the breakfast intervention. The standardized evening meal consisted of Pasta al Pomodora (520 kcal). Study participants were instructed to drink water to the evening meal and arrive fasting to the test kitchen on days of the intervention. All the days during the intervention, study participants noted health status, occasional medications, time for evening meal, and water intake during the overnight fast. The day before and during the intervention, study participants were asked to abstain from eating fish, drinking alcohol, and engaging in strenuous exercise (>2 h moderate intense physical activity, defined as 3–6 MET:s (metabolic equivalents)) [14]. Two weeks before and during the intervention, study participants were also asked to refrain from using dietary supplements and occasional medications. These measures were taken in order to reduce inter- and intra-individual variation.

### 2.4. Sample Collection

Serum was collected before (0 h) and 3 h (190 min ± 11 min) postprandial breakfast meals. In total, 188 samples were collected. Venous blood was drawn into 5 mL BD vacutainer glass tube (BD Hemogard^TM^ BD Vacutainer^®^, Franklin Lakes, NJ, USA). turned approximately five times, allowed to clot at room temperature for 5 min and at 4 °C for additionally 30 min, and centrifuged at 4 °C at 3800 rpm for 10 min. 400 µL serum was aliquoted in 500 µL cryo vials and placed at −0 °C within 1 h (1 h ± 8 min) and at −80 °C within 2 h. Samples were stored at −80 °C until analysis. 

### 2.5. Sample Preparation and NMR Spectroscopy Analysis 

^1^H-nuclear magnetic resonance (NMR) spectroscopy analysis was performed on all of the serum samples. Prior to 1H-NMR analysis, serum samples were thawed for 60 min at 4 °C, 120 µL serum was mixed with 120 µL phosphate buffer (75 mM Na_2_HPO_4_, 20% D_2_O, 0.2 mM imidazole, 4% NaN_3_, 0.08% TSP-d_4_, pH 7.4) in deep well plate and 180 µL transferred to 3.0 mm NMR tubes (Bruker BioSpin, 96 sample racks for SampleJet) using SamplePro (Bruker BioSpin, Rheinstetten, Germany). Samples were kept at 6 °C until analysis. Four pooled serum samples, two Sernorm Human, and two buffer samples were used on each 96 sample rack for quality control. Pooled serum from each breakfasts were used for two-dimensional (2D) ^1^H-^13^C HSQC and ^1^H-^1^H TOCSY experiments.

All ^1^H NMR spectra were measured at 800 MHz using BRUKER Avance HDIII spectrometer equipped with a 3 mm TCI probe and a cooled (6 °C) SampleJet for sample handling. All of the ^1^H NMR experiments were performed at 298 K. NMR data were recorded using the pulse sequence ‘zgespe’, encompassing a perfect echo sequence with excitation sculpting for water suppression. The spectral width was 20 ppm, the relaxation delay was 1.3 s, and the acquisition time was 2.04 s. With a total of 128 scans collected into 64 k data points, the measuring time for each sample was 8 min 9 sec. All of the data sets were zero filled to 128 k and an exponential line-broadening of 0.3 Hz was applied before Fourier transformation. TSP-d4 was used for referencing. ^1^H NMR data was acquired for a total of 188 serum samples. 

For annotation, pooled serum samples, from all the individuals in the dataset, were utilized for natural abundance ^1^H,^13^C HSQC (‘hsqcedetgpsisp2.2’) and ^1^H-^1^H TOCSY (‘mlevgpphw5’) experiments The HSQC spectra were recorded with a 3 s pulse delay, 32 dummy scans, eight scans, and acquisition of 2048 data points (for ^1^H) and 1024 increments (for ^13^C). The ^1^H and ^13^C pulse widths were p1 = 7.4 μs and p3 = 9.3 μs, respectively. The ^1^H and ^13^C spectral widths were 20 ppm and 100.00 ppm, respectively. ^1^H-^1^H TOCSY spectra were acquired with the same proton pulse width as for the HSQC, spectral widths of 14 ppm in both dimensions. The pulse delay was 2 s, 32 scans were recorded and 4096 points and 512 increments were acquired in the direct and indirect dimensions, respectively. All data processing was performed with TopSpin 3.2pl6 (Bruker BioSpin, Rheinstetten, Germany). Sodium phosphate (Na_2_HPO_4_), imidazole, and sodium azide (NaN_3_) were from SigmaAldrich, deuterium oxide (D_2_O) from Cambridge Isotopes and 3-(trimethylsilyl) propionic-2,2,3,3-d_4_ acid sodium salt (TSP-d_4_) from MerckMillipore. Seronorm Human was from Sero A/S. 

### 2.6. Pre-Processing and Statistical Analyses

In total, 14 samples were excluded before data processing and analysis due to poor quality. Hence, 174 samples were further processed. ^1^H NMR spectra were aligned using icoshift [4] and manual integration of peaks was performed to a defined baseline on all spectra in parallel while using an in-house Matlab software application. In total, 196 variables were integrated within chemical shift range of 0.860–8.447 ppm. Prior to modelling, all data were centered and scaled to unit variance. 

Multivariate data analysis (MVA) [6], using principal component analysis (PCA), orthogonal (2) projections to latent structures with discriminant analysis (O2PLS-DA) [15], and orthogonal projections to latent structures with effect projection (OPLS-EP) [16] were performed while using SIMCA software v.15 (Umetrics AB, Umeå, Sweden). 

PCA models were used to explore clustering patterns of observations, trends in the data and outliers. Two different models were generated. One including postprandial (3 h) samples and one including samples that were generated from a calculated effect matrix where the value of each individual and variable at time point 0 h were subtracted from time point 3 h for each breakfast. These models were also used for O2PLS-DA. No outliers were identified in the model, including 3 h samples while three samples were removed as outliers using Hotellings T2 range (T2Crit 99%) and Distance to Model (DModX) (DCrit 0.05) from the effect matrix. In total, 60 and 57 samples were included in the O2PLS-DA models, including 3 h samples and the effect matrix samples, respectively. Samples from individuals lacking a full sample set (samples from all breakfast meals) were used for prediction (Table 1). Five OPLS-EP models were generated. Using the effect matrix that was calculated for the O2PLS-DA model, the individual response from each breakfast meal was investigated, each model including 19 individuals. Additionally, effect matrices were calculated from 3 h samples between the LOV and the VE breakfast and from the LOV and OM breakfasts. Detailed model statistics are presented in Table S5. 

Separation and classification of metabolic profiles between different breakfasts were evaluated using O2PLS-DA. PLS, OPLS, and O2PLS divide the variability of the data into a systematic (R^2^X) and a residual part. In OPLS and O2PLS, the systematic part in turn is divided into a predictive (R^2^Xpred) and an uncorrelated orthogonal (R^2^Xorth) part. The difference between the two is that OPLS only returns one predictive component and O2PLS returns two. When comparing only two groups, OPLS is often used since one component is sufficient to distinguish two groups apart. In the case of three groups, it is reasonable to use two predictive components, allowing for the samples to distribute over a predictive plane rather than a single line. Therefore, O2PLS was used when modelling the VE, LOV, and OM groups jointly in the same model. 

The validity of the O2PLS-DA models were assessed while using default cross validation (every 7th sample), permutation tests (*n* = 999), Coefficient of Variation-Analysis Of Variance testing of Cross-Validated predictive residuals (CV-ANOVA), the cumulative amount of explained variation in the data summarized by the model (R^2^X[cum] and R^2^Y[cum]) and the predictive ability of the model (Q^2^[cum]) visual comparison between scores along the predictive ability of external samples (prediction set). 

OPLS-EP models were used to identify discriminating metabolites between fasting and postprandial samples for each breakfast. Additionally, OPLS-EP models were used to identify the differences in postprandial (3 h) samples between the LOV and VE and between the LOV and OM breakfasts. Prior to modeling, all data were scaled to unit variance. The validity was assessed using default cross validation (every seventh sample), CV-ANOVA, the cumulative amount of explained variation in the data summarized by the model (R^2^X[cum] and R^2^Y[cum]) and the predictive ability of the model (Q^2^[cum]), visual comparison between scores, and the response vector (Y). Discriminating variables were selected using loadings (pq 0.1) and top ranked variables in variable importance (VIP) scores in combination. 

The average fold change for each variable intensity was defined and calculated. Firstly, a vector I0 having the intensity values for each individual at the first time point, and a corresponding vector I3 having the corresponding intensity values at the second time point, were constructed. Secondly, a complex vector Z = I0 + i ∗ I3, where i being the imaginary unit, was calculated. Thirdly, phase angles of each element theta = angle(Z) and the mean of theta, mean(theta) were calculated and mean(theta) defined to be the phase angle of the average fold change giving equal weight for each individuals fold change, irrespective of peak intensities, average fold change = tan(mean(theta)). The reason for using the mean of the phase angles rather than for example the mean of log2 values of the fold changes was to avoid numerical problems, since for some individuals, some measured peak intensities were very small and could not be predicted accurately or had been set to zero. In addition, *p*-values were calculated using the differences of I3-I0 rather than the angles theta, using Wilcoxon signed rank test with Benjamini Hochberg correction at the 95% level. A variable was considered to be significant if *p* < 0.05. These calculations were performed in MatLabR2015a. 

### 2.7. Metabolite Identification

One-dimensional (1D) proton, 2D ^1^H-^13^C HSQC, and 2D ^1^H-^1^H TOCSY spectra of pooled serum from all individuals in the dataset were used for metabolite identification. Chenomx NMR suite 8.1 (Chenomx Inc., Edmonton, AB, Canada) was used for spectral line fitting of 1D proton spectra. Chemical shifts in 1D proton, 2D ^1^H-^13^C HSQC, and 2D ^1^H-^1^H TOCSY spectra were compared with reference spectra/ in the Human Metabolome Database (HMDB) [17].

## 3. Results

**Figure 1:**
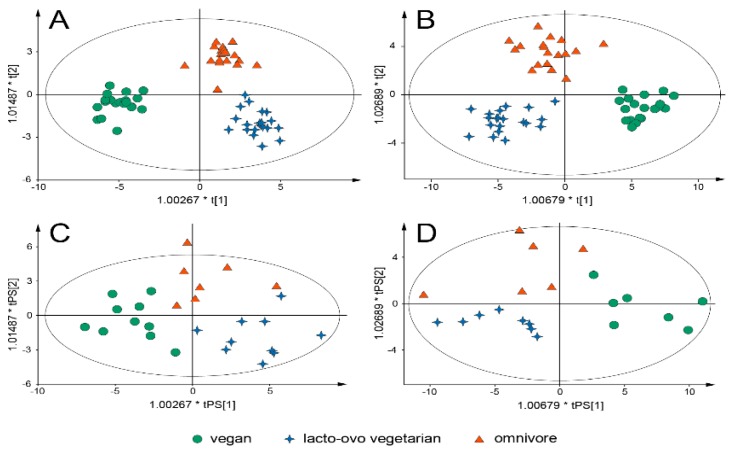
Orthogonal (2) projections to latent structures with discriminant analysis (O2PLS-DA) models of metabolic profiles from vegan, lacto-ovo vegetarian, and omnivore breakfasts. (**A**,**B**) display the distribution of metabolic profiles in O2PLS-DA models and (**C**,**D**) denote samples predicted onto the models. (**A**) (*n* = 60) and (**C**) (*n* = 27) are based on postpandial (3 h) samples. (**B**) (*n* = 57) and (**D**) (*n* = 21) plots are based on an effect matrix where the metabolic profiles have been calculated from the difference between fasting and postprandial state. * The scores are scaled proportionally to their R^2^X values. Both models included 196 variables.

Metabolic profiles from the different breakfast meals, compared using O2PLS-DA models, are displayed in Figure 1. The O2PLS-DA model that is based on the effect matrix classified 90% of samples in the prediction set correctly, while the model that is based on postprandial samples classified 89% correctly (Table S6). However, the model based on the effect matrix resulted in a lower *p*-value and a smaller difference between the R^2^X and Q^2^ values (Table S5).

**Figure 2:**
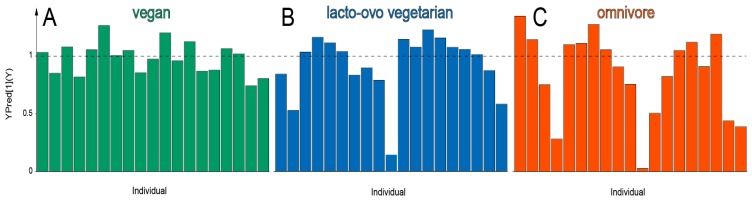
Predicted values in relation to response vector (Y) for volunteers consuming vegan (**A**), lacto-ovo vegetarian (**B**) and omnivore (**C**) breakfasts in orthogonal projections to latent structures with effect matrix (OPLS-EP) models. The dotted line (Y = 1) indicates the response vector value for the models. The magnitude of the predicted effect for each volunteer is given by the height of the corresponding bar. Deviations from the value 1 for a specific volunteer indicate a larger (>1) or smaller (<1) metabolic effect (difference between fasting and postprandial state) in the model direction (metabolic profile) associated with the metabolism of foods included in the different breakfast meals. Each model included 19 observations (equal to number of individuals in the models) and 196 variables. The individual orders of predicted values are not comparable between models.

OPLS-EP models were used to investigate the individual responses for each breakfast meal (Figure 2). All of the individuals displayed a positive response (>0) regarding the effect of the breakfast meals, i.e., fasting samples where distinctly different from postprandial samples. However, the response in one and four individuals where <0.5 after the LOV and OM breakfast, respectively. 

**Figure 3:**
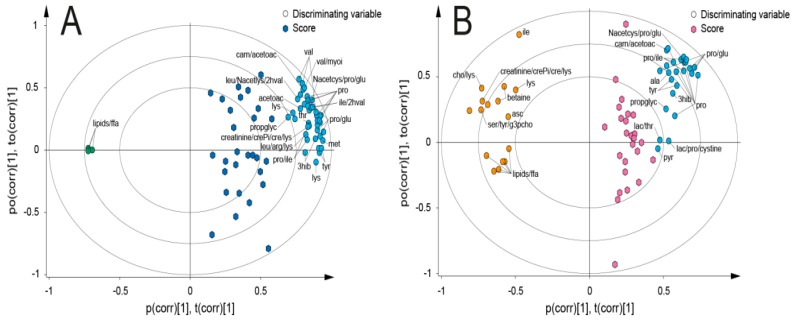
Biplots in orthogonal projections to latent Structures with effect projection (OPLS-EP). Model (**A**) (*n* = 28) compared the difference between lacto-ovo vegetarian (LOV) and vegan (VE) breakfasts. Model (**B**) (*n* = 26) compared the difference between LOV and omnivore (OM) breakfasts. Both models included 196 variables. Scores (hexagon symbols) denote the calculated effect matrix in each individual. Dark blue = difference in metabolic response between LOV and VE. Pink = difference in metabolic response between LOV and OM. Labeled circles denote selected discriminating variables (metabolites) in the two models. Green = metabolites increasing in VE breakfast in relation to LOV breakfast. Blue = metabolites increasing in LOV breakfast in relation to VE and OM breakfasts. Red = metabolites increasing in OM breakfast in relation to LOV breakfast.

In total, the change in concentration of 43 metabolites was identified for each breakfast meal using the effect matrix data (Table S7). To identify the differences in metabolite concentrations between meals, OPLS-EP models were generated where postprandial samples of the LOV were compared to the VE and OM breakfast, respectively (Figure 3). The change in concentration between fasting and postprandial state and between breakfast meals of selected discriminating metabolites in these models is presented in Table 3. Betaine, choline, creatine, isoleucine, and lysine displayed comparably higher concentration in postprandial samples from volunteers who had consumed the OM, in relation to other breakfasts. In turn, carnitine, proline, tyrosine, methionine, and valine showed an increase for both LOV and OM breakfasts between fasting and postprandial state in OPLS-EP models. However, proline, carnitine, and tyrosine were higher in the LOV when compared to the OM breakfast. Alanine and asparagine were detected in a higher concentration after the VE breakfast as compared to fasting samples, but not in relation to the LOV breakfast. In combination, betaine, myo-inositol, and unidentified free fatty acids (FFA) increased for both the VE and OM breakfasts. Postprandial samples from all breakfasts showed a decrease in the concentration of mannose and acetate and an increase in ornithine and glutamate when compared to fasting samples. No difference was seen between breakfasts regarding these metabolites. 

## 4. Discussion

The aim of the present study was to identity postprandial metabolic patterns and metabolites relating to vegan-, lacto-ovo vegetarian-, and omnivore breakfasts in a cross-over design in healthy volunteers. This was achieved using ^1^H NMR metabolomics. We were able to differentiate between metabolic profiles and changes in metabolite concentrations between breakfast meals. In combination, changes in metabolite concentrations between the fasting and postprandial state were identified for all breakfast meals.

Metabolic profiles of the different meals could be characterized using both postprandial samples alone and an effect matrix representative for the metabolic change between fasting and postprandial state. When investigating the individual responses to the different meals, volunteers displayed a homogenous response for the VE, while the responses for the LOV and the OM breakfasts were more heterogeneous, Figure 2. 

The content of the meals was designed to be representative of specific diets, thus the macronutrient distribution was not fixed. The meals contain specific foods that alone or in combination may be responsible for the results seen. Notably, metabolites that were identified in this study may originate from the diet and/or from endogenous processes. In addition, many of these compounds are not exclusively found in only one of these meals or included foods, but the concentration differs and for some metabolites the difference in concentration is reflected in different serum levels. For some metabolites, the difference in serum concentrations identified between meals could be related to the food content. This was the case for choline, betaine, creatine/creatine phosphate, methionine, proline, tyrosine, and branched chain amino acids. 

Choline, betaine, and methionine are involved in betaine metabolism. Betaine is obtained from the diet, either as betaine or choline, which is oxidized to betaine in the human body. Red meat, egg, liver, and soy beans are foods that are rich in choline while high concentrations of betaine are found in wheat products, spinach, beats and shrimps [18]. Here we found increased concentrations of choline and betaine after consumption of the OM when compared to the LOV breakfast, both compared to the fasting state and to the LOV breakfast. 

Methionine is obtained from the diet but can also be generated from homocysteine via betaine. Almonds, sesame seeds, hard cheese, egg, poultry, red meat, sea food, and oat are foods high in methionine. In comparison to the VE breakfast, methionine emerged as a discriminating metabolite for the LOV. Methionine has previously been associated with a western diet high in processed meats, refined grain products, desserts, and sweets [19]. In addition, methionine has also been associated with a diet rich in fish as well as vegetarian diet [20]. Overall, this implies that a meal/diet including animal proteins or proteins from animal derived foods, such as dairy products generates a different increase in methionine in serum, compared to a plant based meal. 

In contradiction to previous results in the literature [21,22], carnitine (in a variable overlapping with acetoacetate) discriminated the LOV from the OM breakfast. Carnitine is found in high concentration in meat products but dairy also contains carnitine, although in lower concentrations [23]. The higher concentration in the LOV breakfast might reflect the rate of metabolism of dairy products as compared to foods that are based on red meat rather than the actual content. Carnitine levels in blood have been shown to be influenced also by other factors, such as the dietary matrix [24], why it is regarded as uncertain as a biomarker of meat intake [25]. About 98% of the dietary creatine is taken up by skeletal muscle, and about 2% of the muscle content is lost daily through conversion to creatinine phosphate, and liberated during the dephosphorylation to form creatinine, which is excreted in urine [26]. Dietary sources of creatine are muscle meats, including fish and only small amounts is found in dairy products. Thus, vegetarians and vegans need to synthesize creatine by de novo biosynthesis from glycine, methionine, and arginine [27]. A variable including creatine, creatinine, and creatinine phosphate was significantly higher in the OM as compared to the LOV and also for the LOV when compared to the VE breakfast, which likely reflects the content of animal derived products from these meals. 

Isoleucine discriminated and increased in concentration after consumption of the OM as compared to the LOV breakfast. Valine increased in concentration for the LOV when compared to the VE breakfast. In turn valine concentrations showed no discriminating difference between the OM and LOV breakfasts in OPLS-EP models, however, increased for both breakfasts in relation to fasting samples. Branched chain amino acids (isoleucine, leucine, and valine) have previously been found in higher concentration in individuals who had consumed an animal protein based diet compared to a vegan diet [10], which is consistent with our results. In combination, 3-hydroxyisobutyrate, which is a by-product of valine catabolism, increased in concentration after both the LOV and OM, although comparably more so for the LOV. 

In comparison of postprandial samples from the LOV and OM breakfasts proline and tyrosine discriminated for the LOV. Dairy products, in particular hard cheese, are high in both proline and tyrosine. Draper et al. have shown a correlation between dietary proline and serum proline concentrations [10]. In addition, postprandial serum tyrosine has previously been reported in higher concentration, following a dairy meal as compared to similar tyrosine content from meals based on fish, meat, and lentils [28]. 

Propylene glycol discriminated for the LOV in the OPLS-EP model, displaying an increase in concentration. Propylene glycol has previously been identified in serum [29]. It is used as a food additive [30] and it has been regarded as an exogenous contaminant [29,31,32]. 

Succinic acid decreased after all breakfasts, although significantly more after the OM when compared to the LOV breakfast. Succinic acid has previously been shown to decrease after a meal containing rye bread [33]. 

Unspecified lipids/FFA were found in increased levels for both the VE and OM breakfasts. The current sample matrix in combination with the present NMR analysis (T2-filtered experiment) negated the identification of individual hydrophobic lipids/FFA. The lipid/FFA fraction of the serum needs to be further investigated to identify individual lipids/FFA and be able to interpret the current findings. 

In a number of studies investigating the metabolome in relation to habitual diet, increased levels of glycine has been shown for vegans [10,20]. However, in our study, the VE breakfast did not produce an increase in glycine as compared to fasting samples or to the LOV breakfast. Glycine has been related to a higher intake of vegetables. In the present study all breakfast meals included vegetables and also fruit in the VE (banana) and LOV (apple) breakfasts, and only the LOV showed a significant change in glycine when compared to fasting samples. This composition of vegetables and fruit might be a potential explanation behind our results. 

Limitations of the present study includes that postprandial samples were collected at only one time point. This limited the potential to investigate the changes in concentration of metabolites over longer time and to capture potential differences as a result of the metabolic turnover of different foods and breakfast meals. Previous studies have shown the concentration of a number of metabolites in blood to peak before 3 h after a challenge meal, especially amino acids and glucose, why sampling at 1 h or 2 h postprandial might have generated different results and amplified knowledge regarding the digestive kinetics of the different meals [11,12,34,35]. The breakfast meals were all equicaloric, but with slightly different macronutrient distribution, due to our aim to use unaltered foods that are representative for each type of breakfast. The OM had the highest protein and fat content and VE the lowest. In comparison, the VE had the highest carbohydrate and fiber content. The macronutrient distribution of our breakfasts meals differed slightly in relation to previous findings regarding the macronutrient intake of vegan-, vegetarian- and omnivore diets [36,37,38]. In addition, difference in macronutrient content of meals can affect both gastric emptying and metabolic responses, such as glucose levels [39,40,41]. The gut microbiota has been shown to change in regard to diet and thus influence the metabolome [42]. As this study only measured the acute effect of one meal this effect was not captured. All volunteers were habitual omnivores and therefore expected to have a different microbiota composition as compared to habitual vegans and lacto-ovo-vegetarians [42]. Still, within the same diet, the individual variation in microbiota composition have been shown to be >70% [43]. In addition, this meal challenge was performed in 32 healthy volunteers, balanced between men and women, and the results might not be generalizable to a more heterogeneous population. 

Strengths of the study include the cross-over design to be able to focus on systematic intra-individual effects of the meal challenge and the standardized evening meal to stabilize the metabolome prior to dietary intervention.

## 5. Conclusions

In the present study, 1H NMR metabolomics showed the ability to differentiate between metabolic profiles from breakfast meals corresponding to three different diets. Changes in metabolite concentrations between meals and between postprandial and fasting state were identified. Our results show that changes in the postprandial concentrations of metabolites in serum can be connected to food consumption. 

## Figures and Tables

**Table 1:** Demographic characteristics of volunteers (*n* = 32).

	Model Set		Prediction Set ^1^	
	Males (*n* = 11)	Females (*n* = 9)	Males (*n* = 5)	Females (*n* = 7)
Characteristics	Mean ± SD	Mean ± SD	Mean ± SD	Mean ± SD
Age (year)	27.0 ± 6.6	25.9 ± 10.1	33.2 ± 13.2	31.9 ± 8.2
Height (cm)	184.1 ± 5.5	168.4 ± 4.7	183.0 ± 4.4	170.9 ± 3.8
Body weight (kg)	79.0 ± 11.0	62.0 ± 5.2	74.5 ± 4.4	60.0 ± 5.4
BMI (kg/m^2^)	23.3 ± 2.5	21.9 ± 1.9	22.2 ± 0.7	20.5 ± 1.6
Fat mass (%)	14.7 ± 5.4	23.5 ± 3.8	15.1 ± 3.0	21.8 ± 5.5

^1^ Samples from individuals lacking a full sample set (samples from all breakfast meals) were used for prediction, described in Section 2.6.

**Table 2:** Meal composition of breakfast meals.

Breakfast ^1^					
Vegan		Lacto-Ovo Vegetarian		Omnivore	
Food	g	Food	g	Food	g
Rye bred	90	Rye bred	90	Rye bred	90
Cashew nut butter	22	Hard cheese 28%	24	Liver pâté	25
Soy yoghurt blueberries	100	Fruit yoghurt 1.7%	100	Smoked ham	30
Olive oil ^2^	2	Cottage cheese 4%	47	Egg	54
Lentils green (dry weight) ^2^	11	Butter and margarine mix 75%	12	Butter and margarine mix 75%	12
Red bell pepper ^2^	9	Apple	20	Red bell pepper	22
Green bell pepper	25	Tomato	25	Cucumber	20
Banana	30	Tea	150	Red caviar	10
Tea	150	Milk (1.5%)	51	Tea	150
Oat milk	50			Milk (1.5%)	51

^1^ Breakfasts including 550 kcal, ratio of content corresponds to breakfasts 750 kcal. ^2^ Included in lentil spread.

**Table 3:** Selected discriminating metabolites in OPLS-EP models.

	Models													
	VE ^1^ Breakfast		LOV ^2^ Breakfast		OM ^3^ Breakfast		LOV vs. VE Breakfast				LOV vs. OM Breakfast			
Characteristic foods	Soy-yoghurt, cashew butter, lentils		Yoghurt, hard cheese, cottage cheese		Liver pâté, smoked ham, egg									
Metabolite	Δconc.	*p*-value ^4^	Δconc.	*p*-value	Δconc.	*p*-value	LOV Δconc.	VE Δconc.	*p*-value	Fold change	LOV Δconc.	OM Δconc.	*p*-value	Fold change
3-Hydroxyisobutyrate			↑	0.0001	↑	0.0004	↑		<0.0001	1.83	↑		0.004	1.28
Acetate	↓	0.0009	↓	<0.0001	↓	0.0002								
Acetoacetate							↑		<0.0001	1.45				
Acetone	↓	0.02												
Alanine	↑	0.0004									↑		0.03	1.11
Glucose (alfa, beta)	↓	0.03	↓	0.0006										
Arginine & Lysine ^5^	↑	0.003	↑	<0.0001	↑	0.0002								
Ascorbate												↑	0.02	0.88
Asparagine	↑	0.02												
Betaine	↑	0.0003			↑	<0.0001						↑	0.02	0.84
Carnitine & Acetoacetate ^5^			↑	<0.0001	↑	0.0002	↑		<0.0001	1.24	↑		0.007	1.18
Choline					↑	0.0001						↑	0.003	0.80
Creatinine	↓	0.009												
Creatinine & Creatine & Creatine phosphate ^5^	↓	0.004					↑		<0.0001	1.12		↑	0.003	0.93
Isoleucine					↑	0.0001	↑		<0.0001	1.21		↑	0.08	0.90
Lactate											↑		0.06	1.22
Leucine	↓	0.05					↑		<0.0001	1.31				
Leucine & Arginine ^5^	↓	0.09	↑	0.002	↑	0.002	↑		<0.0001	1.48				
Lipids/FFA	↑	<0.0001			↑	0.002		↑	<0.0001	0.69		↑	0.003	0.76
Lysine					↑	0.0002	↑		<0.0001	1.51		↑	0.3	0.94
Mannose	↓	0.0004	↓	<0.0001	↓	<0.0001								
Methionine	↓	0.001			↑	0.0002	↑		<0.0001	1.65				
myo-Inositol	↑	0.0007			↑	0.0006								
N-Acetylcysteine & Proline & Glutamate ^5^			↑	<0.0001			↑		<0.0001	1.37	↑		0.005	1.37
O-Phosphocholine & 3-Hydroxybutyrate ^5^	↓	0.005												
Ornithine	↑	0.0001	↑	<0.0001	↑	0.0001								
Proline			↑	<0.0001	↑	0.0001	↑		<0.0001	1.41	↑		0.003	1.29
Proline & Glutamate & Unknown ^5^	↑	0.1	↑	<0.0001	↑	0.0001	↑		<0.0001	1.76	↑		0.003	1.48
Propylene glycol			↑	<0.0001			↑		0.0004	1.42	↑		0.02	1.38
Pyruvate											↑		0.2	1.28
Serine & Tyrosine ^5^												↑	0.001	0.86
Succinic acid	↓	0.0005									↑		0.2	1.26
Threonine	↓	0.004					↑		<0.0001	1.33				
Tyrosine			↑	<0.0001	↑	0.0003	↑		<0.0001	1.42	↑		0.005	1.13
Valine			↑	<0.0001		0.0001	↑		<0.0001	1.22				

^1^ Vegan breakfast Δ in concentration between fasting and postprandial (3 h) state. ^2^ Lacto-ovo vegetarian breakfast Δ in concentration between fasting and postprandial (3 h) state. ^3^ Omnivore breakfast Δ in concentration between fasting and postprandial (3 h) state. ^4^ *p*-value calculated using Wilcoxon signed ranked test. ^5^ Overlapping metabolites. ↓ indicates decreace in concentration of discriminating metabolites in OPLS-EP models. ↑ indicates in concentration of discriminating metabolites in OPLS-EP models

## Supplementary Materials

The following are available online at http://www.mdpi.com/2072-6643/10/8/1063/s1, Table S1: Meal composition of omnivore breakfast, Table S2: Meal composition of lacto-ovo vegetarian breakfast, Table S3: Meal composition of vegan breakfast, Table S4: Nutrients content of breakfast meals, Table S5: Model statistics discriminant analysis and effect projections, Table S6: Classification of prediction set in O2PLS-DA models, Table S7: Change in concentration of identified metabolites between fasting and postprandial samples for vegan-, lacto-ovo vegetarian-, and omnivore breakfasts.
