# Supplementary material for: Nutritional Metabolomics: Postprandial Response of Meals Relating to Vegan, Lacto-Ovo Vegetarian, and Omnivore Diets
Source: Nutrients. 2018 Aug 10;10(8):1063. doi: 10.3390/nu10081063 (PMC6115722; doi:10.3390/nu10081063)
Supplement: Supplementary file 1 [file nutrients-10-01063-s001.zip › nutrients-329163-SI.pdf]

Supplementary material manuscript:

Nutritional metabolomics: Postprandial Response of Meals Relating to Vegan-, Lacto-ovo Vegetarian-, and Omnivore Diets

Millie Rådjursöga[1,*], Helen Lindqvist[1], Anders Pedersen[2], Göran Karlsson[2], Daniel Malmodin[2], Lars Ellegård[1] and Anna Winkvist[1]

[1] Department of Internal Medicine and Clinical Nutrition, Sahlgrenska Academy, University of Gothenburg, Box 459, 405 30 Gothenburg, Sweden; millie.radjursoga@gu.se; helen.lindqvist@gu.se; lasse.ellegard@nutrition.gu.se; anna.winkvist@nutrition.gu.se

[2] Swedish NMR Centre, University of Gothenburg, Box 465, 405 30 Gothenburg, Sweden; anders.pedersen@nmr.gu.se; goran.karlsson@nmr.gu.se; daniel.malmodin@gu.se

* Correspondence: millie.radjursoga@gu.se; Tel.: +46-703-868879

Table S1. Meal composition of omnivore breakfast

	Breakfast size	
Food	550 kcal (g)	750 kcal (g)
Rye bred	90	123
Liver pâté	25	32
Smoked ham	30	41
Egg	54	75
Red bell pepper	22	30
Butter and margarine mix 75%	12	17
Cucumber	20	27
Red caviar	10	14
Tea	150	150
Milk (1.5%)	51	70

Table S2. Meal composition of lacto ovo vegetarian breakfast

	Breakfast size	
Food	550 kcal (g)	750 kcal (g)
Rye bred	90	123
Hard cheese 28%	24	32
Tomato	25	34
Cottage cheese 4%	47	64
Apple	20	27
Butter and margarine mix 75%	12	17
Fruit yoghurt 1.7%	100	136
Tea	150	150
Milk (1.5%)	51	70

Table S3. Meal composition of vegan breakfast

	Breakfast size	
Food	550 kcal (g)	750 kcal (g)
Rye bred	90	123
Banana	30	40
Soy yoghurt blueberries	100	136
Olive oil[1]	2	3
Lentils green (dry weight)[1]	11	15
Red bell pepper[1]	9	12
Green bell pepper	25	35
Cashew nut butter	22	30
Tea	150	150
Oat milk	50	68

[1]Included in lentil spread

Table S4. Nutrients content of breakfast meals

	Vegan		Lacto ovo vegetarian		Omnivore	
Nutrients	550 kcal	750 kcal	550 kcal	750 kcal	550 kcal	750 kcal
Energy (kJ)	2297	3138	2301	3146	2301	3146
Energy (kcal)	549	750	550	752	550	752
Protein (g)	19	26	24	33	26	35
Protein (E%)	14	14	18	18	18	18
Fat (g)	17	23	22	30	26	36
Fat (E%)	28	28	35	35	44	44
Carbohydrates (g)	74	100	60	81	50	68
Carbohydrates (E%)	58	58	47	47	38	38
Fiber (g)	9.9	13.5	6.9	9.4	5.8	7.9

Table S5. Model statistics of discriminant analysis and effect projections models

	Model						
	O2PLS-DA 3h[1]	O2PLS-DA Δ 3h-0h[2]	OPLS-EP VE[3]	OPLS-EP LOV[4]	OPLS-EP OM[5]	OPLS-EP LOV vs VE[6]	OPLS-EP LOV vs OM[7]
No of LV[8]	2+9+0	2+7+0	1+2+0	1+1+0	1+1+0	1+1+0	1+1+0
N samples	60	57	19	19	19	28	26
n samples pred. set	27	21	-	-	-	-	-
n variables	196	196	196	196	196	196	196
R^2X [cum][9]	0.738	0.698	0.495	0.522	0.539	0.497	0.399
R^2Y [cum][10]	0.906	0.878	0.981	0.926	0.85	0.9	0.939
Q^2 [cum][11]	0.567	0.636	0.87	0.84	0.731	0.848	0.832
CV-ANOVA[12] (p-value)	0.02	<0.0001	<0.0001	<0.0001	0.0003	<0.0001	<0.0001
Permutation tests (Q^2)[13]	-0.807	-0.557	-	-	-	-	-

[1]Model including postprandial (3h) samples

[2]Model including samples calculated from an effect matrix (Δ from fasting to postprandial state)

[3]Vegan breakfast

[4]Lacto-ovo vegetarian breakfast

[5]Omnivore breakfast

[6]Lacto-ovo vegetarian vs vegan breakfast

[7]Lacto-ovo vegetarian vs omnivore breakfast

[8]Latent Variables

[9]Cumulative fraction of the sum of squares of X explained by the selected latent variables

[10]Cumulative fraction of the sum of squares of Y explained by the selected latent variables

[11]Cumulative fraction of the sum of squares of Y predicted by the selected latent variables, estimated by cross validation

[12]ANalysis Of VAriance testing of Cross-Validated predictive residuals

[13]The intercept between real and random models, degree of overfit

-Not applicable

Table S6. Classification of prediction set in O2PLS-DA models

	Classification							
	3h[1]				Δ 3h-0h[2]			
True intake	VE[3]	LOV[4]	OM[5]	Correct	VE	LOV	OM	Correct
VE	8			80%	6			86 %
LOV	1	9		90%		8	1	100%
OM	1	1	7	100%	1		5	83%
Total (n)	10	10	7	27	7	8	6	21

[1]Model including postprandial (3h) samples

[2]Model including samples calculated from an effect matrix (Δ from fasting to postprandial state)

[3]Vegan breakfast

[4]Lacto-ovo vegetarian breakfast

[5]Omnivore breakfast

Table S7. Change in concentration of identified metabolites between fasting and postprandial samples for vegan-, lacto-ovo vegetarian-, and omnivore breakfasts.

			Vegan breakfast			Lakto-ovo-vegetarian breakfast			Omnivore breakfast		
Metabolite	Chemical shift (ppm[1])	Level identification[2]	Δconc.	p-value[3]	Fold change	Δconc.	p-value	Fold change	Δconc.	p-value	Fold change
3-Hydroxybutyrate	1.19	2D	↓	0.004	0.56	↓	0.005	0.76	-	0.5	0.91
3-Hydroxyisobutyrate	1.06	1D	-	0.7	0.87	↑	0.0001	1.55	↑	0.003	1.40
Acetate	1.91	2D	↓	0.0009	0.38	↓	<0.0001	0.44	↓	0.0002	0.55
Acetoacetate	2.27	2D	↓	0.04	0.70	-	0.7	0.97	-	0.4	1.10
Acetone	2.22	2D	↓	0.01	0.73	-	1.0	1.02	-	0.7	0.95
Alanine	1.47	2D	↑	0.0004	1.17	↑	0.0009	1.23	↑	0.03	1.13
alfa-Glucose	5.23	2D	↓	0.03	0.91	↓	0.0006	0.91	↓	0.05	0.93
Arginine	1.64	2D	↑	0.0009	1.35	-	1	0.99	-	0.4	1.12
Ascorbate	4.51	1D	-	0.10	1.07	-	1	1.00	↑	0.003	1.14
Asparagine	2.95	1D	↑	0.02	1.14	-	0.4	1.04	↑	0.04	1.10

Betaine	3.26	2D	↑	0.0003	1.17	-	0.1	1.10	↑	<0.0001	1.40
Carnitine & cholines	3.22	2D	↑	0.01	1.05	-	0.07	1.06	↑	0.0004	1.08
Choline	3.19	2D	-	0.6	0.99	-	0.6	1.08	↑	<0.0001	1.37
Citrate	2.51	2D	-	0.7	0.98	-	0.8	0.98	-	1	0.99
Creatine/Creatine phosphate/Creatinine	3.03	2D	↓	0.004	0.88	-	0.5	0.98	↑	0.03	1.06
Creatinine	4.05	2D	↓	0.009	0.92	↓	0.002	0.94	↓	0.02	0.94
Formate	8.45	2D	-	0.8	0.99	-	0.5	0.95	-	0.3	0.93
Glutamate	2.34	2D	↑	0.02	1.24	↑	<0.0001	1.64	↑	0.002	1.33
Glutamine	2.44	2D	-	1	1.00	↑	0.03	1.04	-	0.2	1.04
Glycerol	3.55	2D	-	0.3	0.80	↓	0.02	0.86	↓	0.05	0.85
Glycine	3.55	2D	-	0.7	0.96	↓	0.01	0.92	-	0.5	1.01
Isoleucine	1.00	2D	-	0.8	1.01	↑	0.0006	1.24	↑	<0.0001	1.39
Lactate	4.11	2D	-	0.7	0.89	-	0.6	1.06	-	0.5	0.91
Leucine	0.95	2D	↓	0.05	0.89	↑	0.003	1.15	↑	0.003	1.17
Lysine	302	2D	-	0.1	0.84	↑	0.0009	1.34	↑	0.0002	1.33

Mannose	5.18	1D	↓	0.0004	0.59	↓	<0.0001	0.70	↓	<0.0001	0.77
Methanol	3.35	1D	↑	0.04	1.24	-	0.9	0.96	-	0.2	0.89
Methionine	2.63	2D	↓	0.001	0.75	↑	0.0002	1.28	↑	0.0002	1.29
Methylguanidine	2.81	1D	-	0.9	0.95	-	0.1	1.09	-	0.8	0.91
Methylsuccinate	1.08	1D	-	0.9	0.95	-	0.5	1.06	-	0.3	0.90
myo-Inositol	3.62	2D	↑	0.0005	1.18	↑	0.05	1.09	↑	0.0006	1.20
N-Acetylcysteine	2.93	2D	-	0.7	1.02	↑	<0.0001	1.18	↑	0.001	1.22
Nα-Acetyllysine	2.99	2D	-	0.7	1.01	-	0.6	0.94	-	0.9	1.01
O-Acetylcarnitine	3.18		-	0.1	0.68	-	0.1	0.74	-	0.4	0.93
Ornithine	3.05	2D	↑	<0.0001	1.29	↑	<0.0001	1.32	↑	<0.0001	1.40
Proline	4.13	2D	↑	<0.0001	1.15	↑	<0.0001	1.46	↑	0.0004	1.21
Pyruvate	2.36	2D	-	0.6	0.84	-	1	1.05	-	0.3	0.91
Serine	3.95	2D	-	0.9	0.99	-	0.2	1.06	↑	0.03	1.11
Succinic acid	2.40	2D	↓	0.0005	0.49	↓	0.002	0.64	↓	<0.0001	0.51
Threonine	4.24	2D	↓	0.004	0.78	↑	0.004	1.08	↑	0.04	1.10
Tyrosine	6.88	2D	-	0.9	0.97	↑	<0.0001	1.38	↑	0.0003	1.25

Valine	0.98	2D	-	0.3	1.01	↑	<0.0001	1.21	↑	<0.0001	1.23

[1]parts per million

[2]2D=identification using chemical shift in HSQC and TOCSY spectra compared to HMDB. 1D=identification using ^{1}H NMR spectra in Chenomex.

[3]p-value calculations based on Wicoxon signed rank test comparing the difference between fating and postprandial (3h) samples.

-No statistical significant (p>0.05) change
